# The flexDrive: an ultra-light implant for optical control and highly parallel chronic recording of neuronal ensembles in freely moving mice

**DOI:** 10.3389/fnsys.2013.00008

**Published:** 2013-05-13

**Authors:** Jakob Voigts, Joshua H. Siegle, Dominique L. Pritchett, Christopher I. Moore

**Affiliations:** ^1^Department of Brain and Cognitive Sciences, Massachusetts Institute of TechnologyCambridge, MA, USA; ^2^Department of Neuroscience, Brown UniversityProvidence, RI, USA; ^3^Center for Neuroscience, Boston UniversityBoston, MA, USA

**Keywords:** electrophysiology, microdrive, electrode array, optogenetics, multi-site, free behavior

## Abstract

Electrophysiological recordings from ensembles of neurons in behaving mice are a central tool in the study of neural circuits. Despite the widespread use of chronic electrophysiology, the precise positioning of recording electrodes required for high-quality recordings remains a challenge, especially in behaving mice. The complexity of available drive mechanisms, combined with restrictions on implant weight tolerated by mice, limits current methods to recordings from no more than 4–8 electrodes in a single target area. We developed a highly miniaturized yet simple drive design that can be used to independently position 16 electrodes with up to 64 channels in a package that weighs ~2 g. This advance over current designs is achieved by a novel spring-based drive mechanism that reduces implant weight and complexity. The device is easy to build and accommodates arbitrary spatial arrangements of electrodes. Multiple optical fibers can be integrated into the recording array and independently manipulated in depth. Thus, our novel design enables precise optogenetic control and highly parallel chronic recordings of identified single neurons throughout neural circuits in mice.

## Introduction

Neuroscience increasingly relies on ensemble recordings that characterize not only individual neurons, but also the complex interplay of neurons within local circuits and across different brain areas (Miller and Wilson, 2008). Recently, the development of optogenetic tools (Boyden et al., 2005; Zhang et al., 2007; Cardin et al., 2010; Kravitz and Kreitzer, 2011; Pastrana, 2011, for a primer) facilitated the precise, cell-type specific optical manipulation of neural circuits in behaving animals. The availability of transgenic mouse lines that allow the expression of light-gated ion channels in specific cell types makes it especially desirable to probe the interactions between cell types in neural circuits with large simultaneous recordings in behaving mice.

While the use of large-scale recordings in behaving animals has been highly successful in primates (Serruya et al., 2002; Nicolelis et al., 2003; Lebedev and Nicolelis, 2006; Buschman and Miller, 2007; Feingold et al., 2012), and to some degree in rats (Nicolelis et al., 1993), adapting the approach to mice has been difficult due to their smaller size. During experiments, implant weight can be offset by a pulley system with a counter weight (Yamamoto and Wilson, 2008) or attaching a helium-filled balloon to the implant (Lin et al., 2006), or by placing the animal in a headpost (Dombeck et al., 2007). However, a mouse's comfort and post-op survival depends critically on its ability to move, eat, and drink in its home cage, imposing a weight limit of ~4 g on implants. For applications that combine recording with behavioral phenotyping (Crawley, 2007), an implant weight closer to 2 g is required so that the mice can move freely or so that the implant can be tilted in a way that does not occlude the field of view in experiments requiring videography (Ritt et al., 2008; Voigts et al., 2008). Studies that address the distributed development of neural systems in adolescent animals also require lighter implants.

One approach to minimizing implant weight while keeping channel count high is to use static electrode arrays, thereby relieving the added weight of multiple independent drives (Bragin et al., 2000). Alternatively, large numbers of electrodes can be slowly lowered into the brain with a single drive mechanism or in a few individually movable groups. This approach has been shown to result in high unit yields for cortical or hippocampal recordings either using arrays of micro-wires (Lin et al., 2006) or laminar silicon probes (Vandecasteele et al., 2012).

While useful for many applications, there are some drawbacks to static implants, or implants that don't allow adjustment of individual electrodes. First, if drive placement is inaccurate initially, electrode position cannot be corrected, or requires changing all recording sites at once. Second, a more subtle but equally important concern is the need to move electrodes to continue to obtain high-quality units. One of the main constraints on the duration that any electrode can yield high quality single-unit data is gliosis, the process of successive encapsulation of foreign materials by glial cells that insulate the electrode from surrounding neurons (Turner et al., 1999; Polikov et al., 2005). Even though stable recording conditions can be maintained over months in optimal conditions (Freire et al., 2011; Tseng et al., 2011), the process of glial encapsulation begins as early as one day post-implant (Fujita et al., 1998) and can lead to a progressive deterioration in the experimenter's ability to identify and discriminate individual neurons (Williams et al., 1999; Vetter et al., 2004; Dickey et al., 2009; Muthuswamy et al., 2011). Similarly, small movements of electrodes relative to the surrounding tissue can damage the neuropil and lead to a decline in unit yield over time.

Consequently, obtaining and maintaining high-quality recordings over many days requires the ability to precisely reposition the recording electrodes in the neural tissue long after the initial implant surgery. By lowering electrodes after the date of implantation, their recording sites can be repeatedly moved out of the zone of neural degradation or glial migration (Jackson et al., 2010; Muthuswamy et al., 2011). (See Figure 3C for an example where lowering an electrode by ~60 μm restores the recording quality of a stereotrode that previously lost the ability to resolve units.), Such motions also make it possible to delay the onset of the electrode-related tissue reactions altogether until after the completion of behavioral training, for example. While there are mouse-specific commercially available implants with up to 8 individually movable electrodes (VersaDrive, Neuralynx, Bozeman MT; Jog et al., 2002; see Dobbins et al., 2007 for protocol), as well as custom-built designs that can combine optical fibers with tetrodes (Fee and Leonardo, 2001; Korshunov, 2006; Lansink et al., 2007; Kloosterman et al., 2009; Haiss et al., 2010; Anikeeva et al., 2012), no current method combines the key features of low weight, high number of individually movable electrodes, high placement stability, and independently adjustable optical fibers. These features are essential for obtaining high-quality, parallel, and distributed recordings within and across neural circuits while providing precise optogenetic control of target neurons. While solutions based on microscale motors are starting to become feasible (Muthuswamy et al., 2011), it will be several years before they surpass the established microwire electrode methods in terms of channel count, availability of multi-site electrodes, ease of use, and cost.

**Figure 1:**
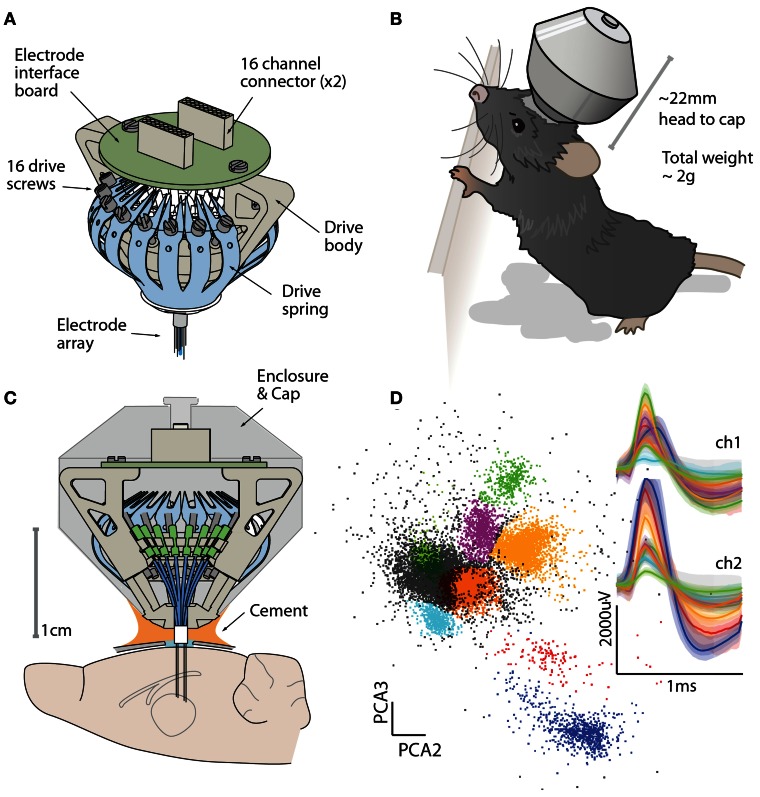
**The flexDrive provides a low-weight and high-yield method for chronic electrophysiology. (A)** Isometric view of the flexDrive showing the one-piece spring (blue) that acts as the drive mechanism. **(B)** Illustration of the flexDrive implanted in a 6 month old C57/bl6 mouse. Due to the low implant weight (~2 g), the impact of the drive on natural behavior is minimal. **(C)** Cross section of the drive and its placement on the mouse skull. In this example, electrodes target the thalamus. **(D)** Cortical action potentials recorded from a stereotrode (12 μm nichrome wire, gold plated to ~300 KΩ) on a flexDrive showing eight clusters (color coded clusters, non-clustered spikes in gray) and average and 95% percentiles of the waveforms on the two electrode contacts.

To overcome these limitations in current methods, we have developed a simple, highly miniaturized drive design that replaces the drive mechanisms found in current implants with a one-piece spring design. This results in a significant reduction in drive size and weight, without sacrificing channel count. Our “flexDrive” fits up to 16 individually movable electrodes or electrode bundles and can maintain stable recording conditions for months. The design integrates guides for two or more optical fibers, which can either be static or adjustable. Due to its small size and low weight (~2 g, ~2 cm height, ~1.5 cm diameter, Figures 1B,C), the flexDrive is well tolerated by mice with only minimal impact on natural behavior.

The design includes support for 16, 32, or 64 channels and interfaces with standard amplifier connectors. The drive can be assembled in about one day after little training, and can be customized to fit specific experimental requirements such as different electrode types, spatial arrangement of the electrodes and optical fibers, or amplifier interface. The custom-made parts of the flexDrive can be ordered from online vendors or workshops with the provided design specifications. All design files are available under the TAPR Open Hardware license, which requires others to make adaptations of the design freely available as well (see http://neuroscience.brown.edu/moore/ and http://github.com/open-ephys/flexDrive).

The design described here presents a significant improvement in the quality and quantity of the data that can be obtained in experiments using optogenetic circuit manipulations in mice, enabling the study of the concerted function of large neural circuits, rather than local neurons.

## Materials and methods

### Electrode array patterns

**Figure 2:**
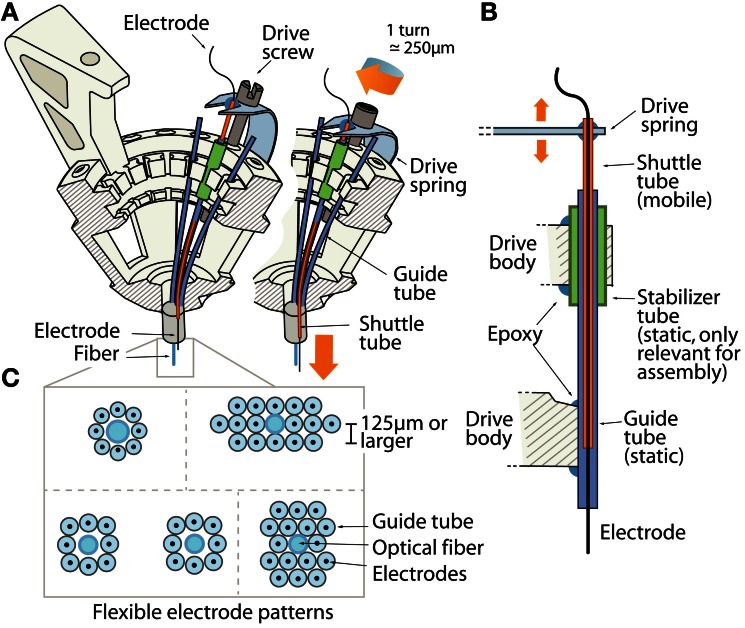
**The drive mechanism of the flexDrive. (A)** Isometric view of the spring loaded drive mechanism. The pattern of electrodes is defined by an array of guide tubes (blue). Electrodes (black) are fixed inside shuttle tubes (orange) that can move up or down inside the guide tubes. The top of each shuttle tube is glued to a spring arm that is moved up or down by a drive screw. **(B)** Schematic view of the drive mechanism (not to scale). The static guide tubes (blue, part of the guide tube array) house the mobile shuttle tubes (orange) that are moved by the drive spring. Stabilizer tubes (green) are used to facilitate assembly of the guide tube array. **(C)** Examples of electrode patterns that can be fabricated by arrangement of the guide tubes and optical fibers.

Given that large-scale recordings of neural activity rely on precise positioning of many electrodes, we designed the flexDrive around a method that allow the experimenter to arrange electrodes in a variety of patterns. In our design, electrodes are positioned by an array of flexible polyimide tubes. By placing individual guide tube arrays at different locations within the drive body, multiple brain regions can be targeted precisely. This control gives researchers the ability to adapt the design to fit their specific experimental needs, such as recording from elongated but narrow target regions (or from bilateral targets) with a single implant (Figures 2A,C).

The array of guide tubes is assembled by building up layers of polyimide tubes and fixing them with cyanoacrylate glue (see Supplementary Material). Electrodes can either be layered in an offset pattern with each layer resting in the grooves of the preceding layer, resulting in a “honeycomb” type pattern, or layered with no offset giving a rectangular pattern (Figure 2C). Alternatively, arranging the guide tubes within a larger guide cannula can make this process faster, but sacrifices some flexibility. By using only a subset of the guide tubes to hold electrodes, or by introducing placeholders and optical fibers into the array, any spatial pattern of electrode and optical fiber positions can be fabricated with high repeatability and precision. The electrodes are free to move laterally within the guide tubes. Such laterally flexible anchoring of electrodes has been shown to decrease adverse tissue reactions (Biran et al., 2007).

The closest lateral spacing between electrodes that can be accomplished with this method is dictated by the outer diameter of the guide tubes. We recommend a distance of ~250 μm or larger for the guide tubes (using 33 gauge), but higher densities of ~125 μm are possible by using smaller diameter guide tubes. However, tests conducted with dense electrode arrays of pitches of 125 μm failed to yield usable recordings, possibly due to an increased inflammatory response.

The array of guide tubes is attached to a plastic drive body (Figures 1A, 2A) that is manufactured from an Acrylonitrile butadiene styrene (ABS)-like material using stereolitography (Accura55 American Precision Prototyping, proprietary material). This drive body supports all components of the drive and facilitates fast and precise assembly. While most components are eventually fixed with an epoxy glue, the design features “snap-fit” grooves, facilitating the alignment of the guide tubes and the spring. By customizing the locations of the guide tubes in the drive body Computer aided design (CAD) file, precise targeting of separate recording sites are readily achieved.

### Drive mechanism

A central constraint on data collection in chronic electrophysiology is the difficulty of recording the activity of identified, individual neurons (termed “units”). While the use of tetrodes (Wilson and McNaughton, 1993; Gray et al., 1995; Jog et al., 2002; Nguyen et al., 2009) or stereotrodes (McNaughton et al., 1983) have made it possible to reliably identify individual neurons in recordings and to record from the same neurons over consecutive sessions (Tolias et al., 2007), obtaining sufficiently clear data from large numbers of electrodes remains a challenge. The presented drive design addresses this constraint by enabling highly parallel recordings in mice without sacrificing the ability to precisely reposition many individual electrodes.

To enable the precise positioning and re-positioning of electrodes in the awake mouse brain, we replaced the traditional multi-part drive design (Kloosterman et al., 2009) with a simplified mechanism in which a single spring and one screw per electrode form the adjustment mechanism. Each electrode (or electrode bundle) is inserted through a piece of polyimide tubing, called a “shuttle tube,” that can move up and down in its guide tube. The linear motion that drives the electrodes is provided by the 16 arms of a single steel spring that are each held down by an adjustment screw (Figure 2A). Each shuttle tube is attached to one of the arms of the spring. By turning the screw, the spring arm is either pressed down or released, which translates to a linear motion of the shuttle tube within the guide tube, moving the electrode in the brain. Due to the inherent stiffness of microwire electrodes, the electrodes move in straight tracks once they exit the guide tubes (Jog et al., 2002; Dobbins et al., 2007; Lansink et al., 2007).

**Figure 3:**
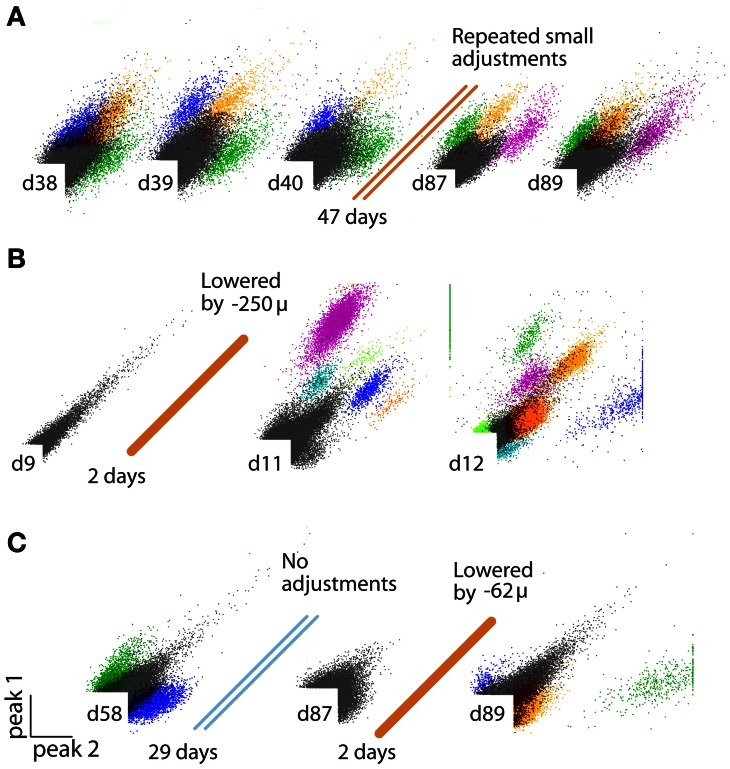
**(A–C)** Examples of identified units on stereotrodes, all plots peak/peak. **(A)** Recording quality sufficient for sorting units can be maintained on an electrode for >100 days by repeated small increments in electrode depth. **(B)** Example of an electrode that was not penetrating the cortex at surgery, but is lowered into the brain later. **(C)** Example of an electrode that loses the ability to discriminate units over time, but is “reactivated” by a small depth adjustment ~3 months after surgery.

Each turn of a screw corresponds to ~250 μm in electrode motion. The tension and lateral stability of the spring arm ensures that there is no sideways travel or twisting of the shuttle tube when the screw is adjusted, and allows the electrode to be moved smoothly both up and downwards. By adjusting the drive screws in small increments, fine grained control over electrode depth is possible. In practice, we found that quarter turns (~62 μm) are a useful step size that can be used to recover recording quality on electrodes that have lost the ability to resolve units due to tissue degeneration (Figure 3C). This step size is larger than the minimal movement required for recovering units (Yamamoto and Wilson, 2008) but is easy to achieve by manual adjustment. When lowering electrodes to deep targets, we found that adjustments of half turns (~125 μm) every 2–4 days result in stable recording conditions, but the protocol for lowering the electrodes has to take into account the specific type of electrode in use, as well as the target site and aim of the experiment.

Implant fabrication is simplified by this spring loaded drive design (see Supplementary Material). The array of guide tubes is glued to the drive body and individual tubes snap-fit into prepared grooves that ensure proper alignment. This step of attaching the guide tubes to the drive body is simplified by a feature in the drive body design that allows users to temporarily fix the guide tubes in their grooves without glue by sliding a short piece of polyimide tubing (“stabilizer tube,” see Figures 2A,C) over the guide tube, thereby holding it in place for gluing.

The spring is then attached in one step and its arms are moved under the screw heads. The shuttle tubes are then glued to these spring arms, completing the construction of the drive mechanism and making the drive ready for loading with electrodes.

By making it possible to individually adjust electrode depth, this design facilitates the targeting of small target regions, enables significantly higher unit yields over longer time spans than previously possible, and enables highly parallel recordings in awake, behaving mice

### Optical fibers

Experiments using optogenetic manipulation of neural circuits often require spatially distinct recording and stimulation sites. Current approaches such as integrating optical fibers into arrays of silicon probes (Royer et al., 2010) or attaching tetrodes to optical fibers (Anikeeva et al., 2012) provide a very high spatial precision in the relative position between light source and recording sites but don't provide the ability to adjust their relative position after the surgery.

**Figure 4:**
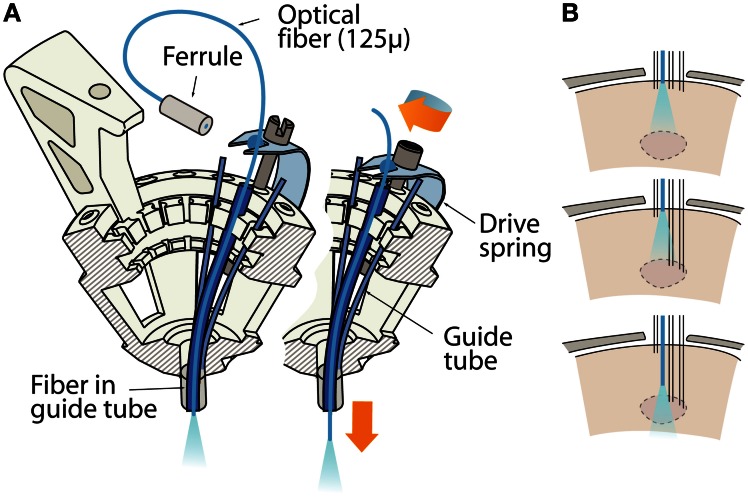
**Variant of the flexDrive in which an optical fiber is lowered in the brain by one of the 16 drive mechanisms. (A)** The fiber is inserted through a guide tube and fixed to a drive spring, replacing a shuttle tube and electrode. The remaining 15 drives can be used for electrodes or more fibers. **(B)** Sketch of the workflow of an experiment made possible through moveable fibers and electrodes. A target area (dashed lines) is localized by slowly lowering a subset of electrodes first, then the fiber can be brought into optimal position for localized activation of the area or for the collection of optical signals.

In the flexDrive, optical fibers can be built into the guide tube array at any desired position and depth (Figure 2C) and can remain static while any of the surrounding electrodes are lowered (Figure 2A). Such an arrangement limits the deformation of brain tissue during electrode adjustment compared to methods in which fibers and electrodes move together (Anikeeva et al., 2012), and ensures that optical stimulation parameters will be reproducible throughout the entire experiment. However, if independent adjustment of the fiber depth throughout the experiment is desired, one or more small diameter fibers (~125 μ) can be inserted in place of electrodes and can be lowered using the same spring-driven mechanism (Figure 4). In this case, the fiber is lowered into a guide tube and glued to the spring in place of a shuttle tube. The free upper end of the fiber with the ferrule connector (extending ~2 cm past the spring) is then looped around and fixed perpendicular to the electrode interface board using epoxy (Figure 4A). This free loop provides enough flexibility for the fiber to move up and down. If desired, electrodes can be glued to the fibers at constant depth offset (Anikeeva et al., 2012). In practice, we find that attaching 2 ferrules to the electrode interface board is straightforward, though in principle up to 16 fibers could be attached.

This variant of the flexDrive enables researchers to precisely position optical fibers to electrophysiologically identified target areas (Figure 4B), or to compare optical manipulation of neural activity in different positions in the same animal. Further, this method enables the collection of optical signals from fluorescent probes (O'Connor et al., 2009; Scanziani and Häusser, 2009) while simultaneously recording extracellularly. Similarly, other sufficiently flexible probes such as microdialysis tubes or voltammetry probes could be added to the recording array.

## Results

### Recording from optically activated identified neurons and distributed, small targets

**Figure 5:**
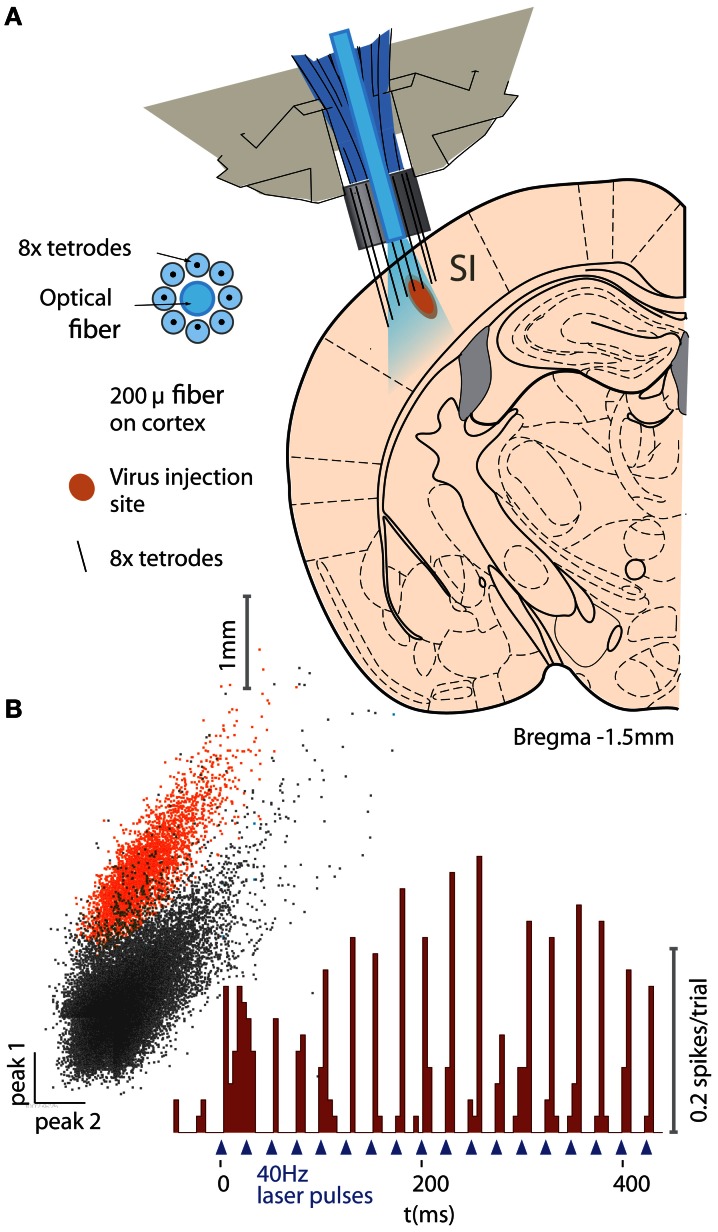
**Example application of the flexDrive for an experiment that require stable optical excitation of neurons. (A)** Activation of PV-positive neurons in layer 2/3 of mouse primary somatosenory cortex (SI) with ChR2. An array of 8 tetrodes arranged in a circular pattern around a static 200 μm fiber (see insert) were slowly lowered into layer 2/3 of SI. **(B)** Example trace of an identified PV neuron on one of the tetrodes for one session.

To verify the utility of our drive design for optical activation of neurons in a chronic behaving mouse, we implanted parvalbumin (PV)−Cre+ mice with a double-floxed adeno-associated carrying the gene for channelrhodopsin-2 (ChR2) (Cardin et al., 2009, 2010; Wang and Carlén, 2012). All experimental procedures were in accordance with the guidelines published in the National Institutes of Health Guide for the Care and Use of Laboratory Animals and were approved by the Brown University Institutional Animal Care and Use Committee. The virus was injected into the barrel field of the primary somatosensory cortex (SI) during the same surgical procedure in which the flexDrive was implanted. Due to injection depth, the virus was expressed predominantly in the fast-spiking interneurons in cortical layers 2/3 and 4, as confirmed by post-mortem histology. We implanted the mice with drives constructed with 8 tetrodes surrounding a static 200 μm optical fiber (Figure 5A).

Electrophysiological signals were recorded with a Plexon Recorder system (Plexon, Dallas TX) and a Neuralynx Cheetah system (Neuralynx, Bozeman MT), bandpass filtered (300–9000 Hz) and spikes were detected using manually set thresholds. Spikes were clustered based on waveform features across the contacts of the stereotrodes or tetrodes using custom software (available at http://moorelab.github.com/simpleclust). Spike trains were designated as “single unit” recordings if their waveforms were clearly separated from other waveforms, agreed with the expected waveform for units in the target area (Cardin et al., 2009; Halassa et al., 2011) and if the distribution of inter-spike-intervals showed a refractory period.

The tetrodes were lowered individually while the fiber remained fixed on the surface of the neocortex. By adjusting the depth of individual electrodes, we were able to record neurons in SI (Figures 5A,B) over the span of the experiment (~3 months) with an average yield of 2.50 cells/tetrode resulting in 20 ± 4.7 simultaneously recorded units (*N* = 8 tetrodes in the target region over 17 sessions).

This approach presents a marked improvement in the quality of data compared to prior experiments in which we used static electrode implants.

**Figure 6:**
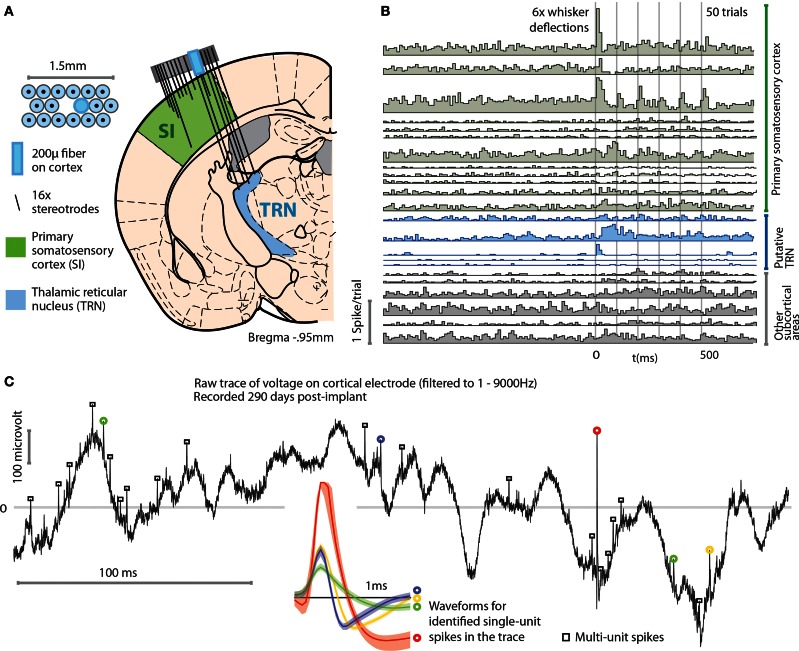
**Example application of the flexDrive for an experiment that requires simultaneous recordings from distributed, small target regions. (A)** Experiment in which an array of 16 stereotrodes was used to simultaneously record from SI and the thalamic reticular nucleus (TRN) in awake, behaving mice. The electrode positions are shown for the 3rd day after the first electrodes reached TRN. **(B)** Example peri-stimulus time histograms of 23 simultaneously recorded single units. A subset of the recorded neurons in SI and TRN are modulated by vibrissa deflections induced with a piezoelectric stimulator. **(C)** Example voltage trace (bandpass filtered at 1–9000 Hz) from a cortical electrode 290 days after the implant surgery. Colored circles and spike waveforms show spikes from 4 identified single units.

In a separate experiment, we implanted mice with flexDrives designed to target both SI and the thalamic reticular nucleus (TRN) (Figure 6A). The TRN presents a thin target, and recording units from this brain region has proven challenging in the past (Halassa et al., 2011). Using the flexDrive, we were able to slowly advance electrodes until the electrophysiological signature of the recording indicated that we reached TRN (identified by elevated tonic firing rate and thin spike waveforms—Pinault, 2004; Halassa et al., 2011). In these target regions, our method resulted in a unit yield of 1.43 cells/stereotrode (*N* = 32 electrodes over 16 sessions each) which enabled recordings from ~10–20 units per recording session (Figure 6B). The drive implant allowed us to place some electrodes with very good longevity, with some channels yielding well separated single units up to 290 days post-implant (Figure 6C).

As shown by these examples, the precise positioning of 16 individual multi-contact electrodes afforded by the flexDrive allows us to record high-yield spike trains from identified single neurons in small target regions such as TRN.

## Discussion

Combining high density parallel recordings of identified neurons throughout neural circuits with the specificity of optogenetic control is essential for experiments seeking to understand complex neural circuits. Recently, studies have demonstrated the great utility of simultaneous optogenetic interventions and single- and multi-unit recordings from awake, behaving mice (Halassa et al., 2011; Tye and Deisseroth, 2012). However, these studies lacked the ability to record neural activity from more than a few identified neurons at a time, mainly due to the use of static electrodes. In addition, recordings of identified neurons across multiple brain regions have been limited by the complexity and weight of the required implants.

**Figure 7:**
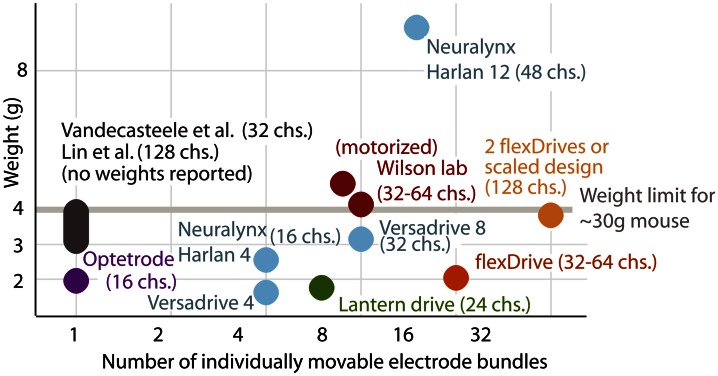
**Comparison between existing types of implants and the flexDrive.** Our novel design results in a higher number of individually movable electrodes at a reduced implant weight compared to existing methods (Lin et al., 2006; Yamamoto and Wilson, 2008; Battaglia et al., 2009; Kloosterman et al., 2009; Anikeeva et al., 2012; Vandecasteele et al., 2012; Neuralynx-Bozeman MT). The drive weight of ~2 g enables experimenters to either implant two drives per mouse, or to scale the design to 32 driven electrodes per implant.

Here, we described the design of a drive implant that provides the ability to record from 16 individually adjustable multi-contact electrodes simultaneously for months in awake, behaving mice while optogenetically manipulating neural activity. By replacing the complex mechanisms employed in previous drive designs with a simple spring design (Figures 1, 2) the flexDrive is significantly lighter (Figure 7) and easier to construct. The ability to independently adjust each electrode over months (Figures 2, 3) allows for high flexibility in recording from small target areas and results in higher yields of well-isolated single-unit activity, over significantly longer time spans than would be possible with static implants.

By repeatedly lowering electrodes, multiple attempts at obtaining good recordings can be made. This is especially important when targeting specific cell types in small target regions (Figure 6), or thin laminated structures such as the cell layers of the hippocampus (Kloosterman et al., 2009), or specific cortical layers (Figure 5). Delaying the lowering of electrodes until after surgery also increases the targeting precision because electrodes can be positioned after initial brain swelling has subsided (Cole et al., 2011). This procedural step is of increased importance for the large craniotomies required for distributed recordings over more than one target site. Finally, implantation of large electrode arrays with pitches below 250 μm raises the risk of brain deformation during insertion due to the increased localized friction between the electrodes and brain tissue (Rennaker et al., 2005). This problem can be mitigated by individually lowering the electrodes one at a time.

We have demonstrated the ability of the flexDrive to record from light-driven PV-positive interneurons in layers 2/3 of SI barrel cortex of awake mice using ChR2 (Figure 5). The light was delivered to the neurons through an optical fiber positioned on the pial surface, showing the ability of the drive to record from optically driven neurons with independent positioning of the recording electrodes relative to the light source. We further demonstrated the utility of the design by recording simultaneous single neurons from SI and the TRN (Figure 6) showing that the design can combine highly parallel recordings from 16 electrodes with the positioning accuracy required to observe neurons in small, deep targets such as the TRN.

To conclude, the flexDrive presents a straightforward method for obtaining stable and high-quality electrophysiological data from multiple target sites in awake, behaving mice. This permits researchers to make full use of the precision and specificity of optogenetic methods by directly probing the concerted function of neural circuits, rather than individual neurons.

### Conflict of interest statement

The authors declare that the research was conducted in the absence of any commercial or financial relationships that could be construed as a potential conflict of interest.
