# Supplementary material for: The flexDrive: an ultra-light implant for optical control and highly parallel chronic recording of neuronal ensembles in freely moving mice
Source: Front Syst Neurosci. 2013 May 13;7:8. doi: 10.3389/fnsys.2013.00008 (PMC3652307; doi:10.3389/fnsys.2013.00008)
Supplement: Supplementary file 1 [file DataSheet1.ZIP › flexDrive_source_files_mar11/spring_16drives/flexDrive_spring_16drives_rev1_0_drawing.PDF]

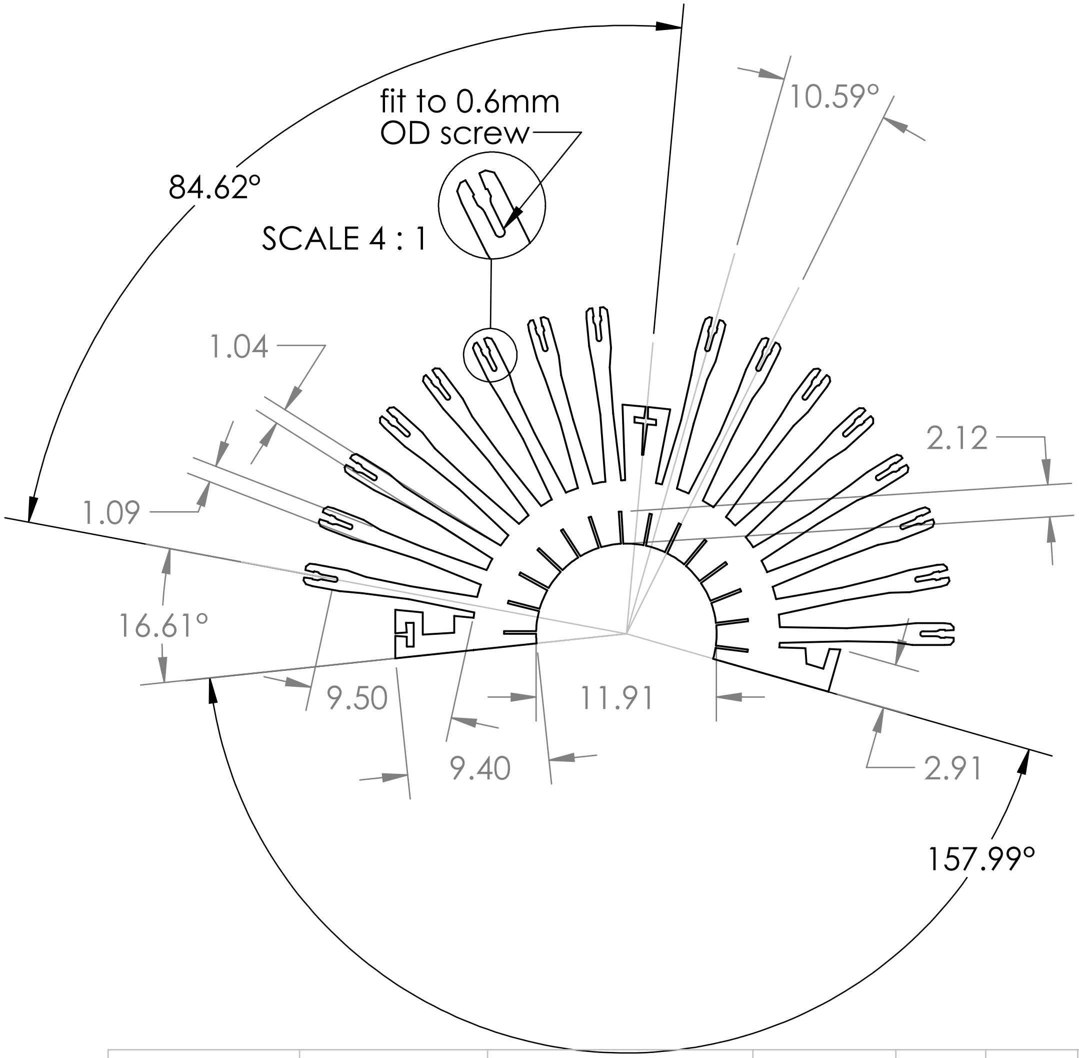

		UNLESS OTHERWISE SPECIFIED:		NAME	DATE
		DIMENSIONS ARE IN INCHES<br>TOLERANCES:<br>FRACTIONAL ±<br>ANGULAR: MACH ± BEND ±<br>TWO PLACE DECIMAL ±<br>THREE PLACE DECIMAL ±	DRAWN		
			CHECKED		
			ENG APPR.		
			MFG APPR.		
		INTERPRET GEOMETRIC TOLERANCING PER:	Q.A.		
		MATERIAL	COMMENTS:		
NEXT ASSY	USED ON	FINISH			
APPLICATION		DO NOT SCALE DRAWING			

TITLE:

SIZE | DWG. NO. | REV

flexDrive_spring_16drives_re

SCALE: 2:1 | WEIGHT: | SHEET 1 OF 1

PROPRIETARY AND CONFIDENTIAL

THE INFORMATION CONTAINED IN THIS DRAWING IS THE SOLE PROPERTY OF <INSERT COMPANY NAME HERE>. ANY REPRODUCTION IN PART OR AS A WHOLE WITHOUT THE WRITTEN PERMISSION OF <INSERT COMPANY NAME HERE> IS PROHIBITED.

5 4 3 2 1
