# Supplementary material for: The flexDrive: an ultra-light implant for optical control and highly parallel chronic recording of neuronal ensembles in freely moving mice
Source: Front Syst Neurosci. 2013 May 13;7:8. doi: 10.3389/fnsys.2013.00008 (PMC3652307; doi:10.3389/fnsys.2013.00008)
Supplement: Supplementary file 1 [file DataSheet1.ZIP › flexDrive_source_files_mar11/drive_body/flexDrive_base_16drives_rev1_1_drawing01.PDF]

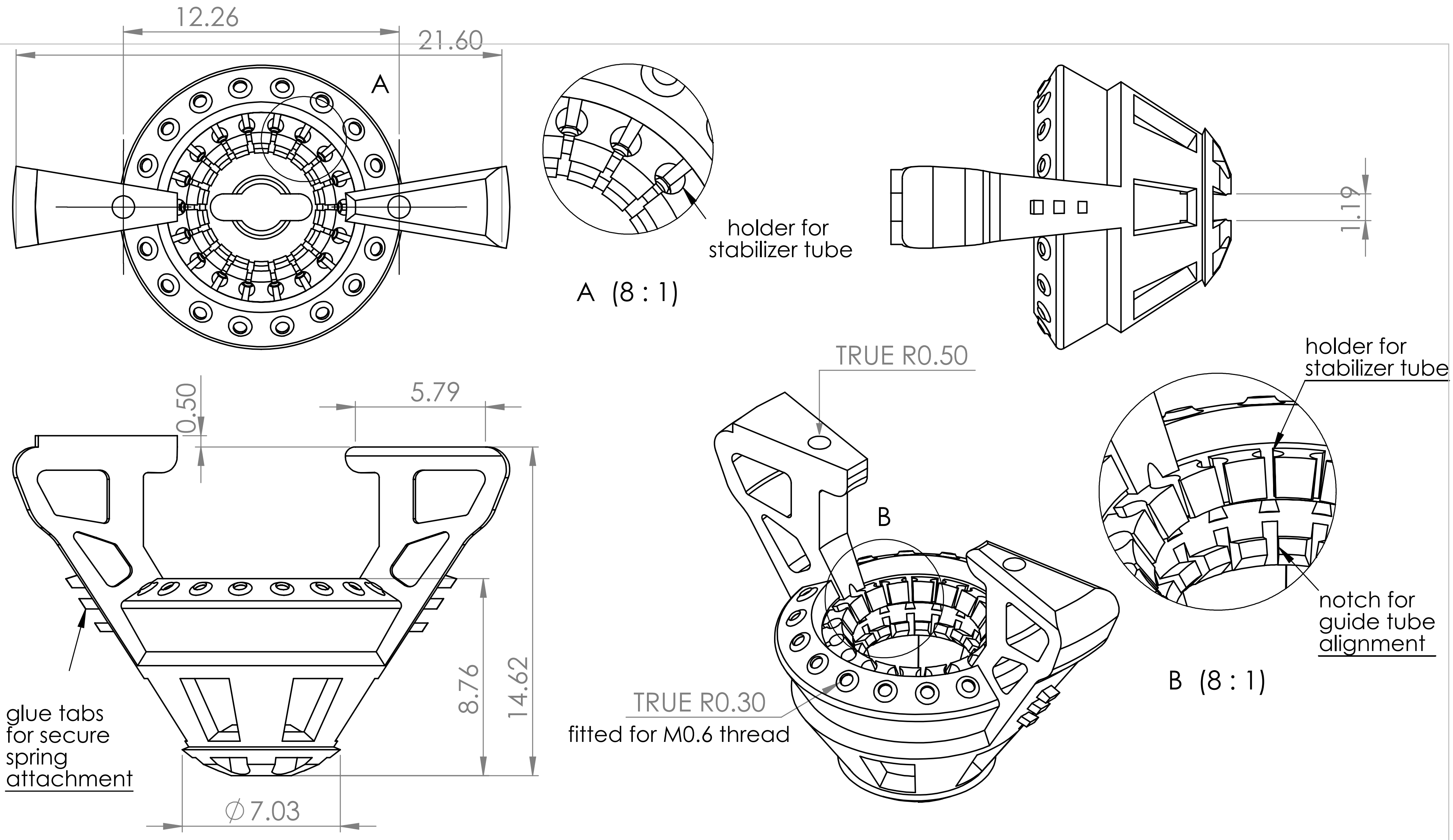

PROPRIETARY AND CONFIDENTIAL

THE INFORMATION CONTAINED IN THIS DRAWING IS THE SOLE PROPERTY OF <INSERT COMPANY NAME HERE>. ANY REPRODUCTION IN PART OR AS A WHOLE WITHOUT THE WRITTEN PERMISSION OF <INSERT COMPANY NAME HERE> IS PROHIBITED.

NEXT ASSY	USED ON
APPLICATION	

UNLESS OTHERWISE SPECIFIED:

DIMENSIONS ARE IN INCHES
TOLERANCES:
FRACTIONAL ±
ANGULAR: MACH ± BEND ±
TWO PLACE DECIMAL ±
THREE PLACE DECIMAL ±

INTERPRET GEOMETRIC TOLERANCING PER:

MATERIAL

FINISH

DO NOT SCALE DRAWING

	NAME	DATE
DRAWN		
CHECKED		
ENG APPR.		
MFG APPR.		
Q.A.		

COMMENTS:

TITLE:

SIZE A | DWG. NO. flexDrive_base_16drives_rev | REV

SCALE: 4:1 | WEIGHT: | SHEET 1 OF 1

5 4 3 2 1
