# Supplementary figures and images for: The flexDrive: an ultra-light implant for optical control and highly parallel chronic recording of neuronal ensembles in freely moving mice
Source: Front Syst Neurosci. 2013 May 13;7:8. doi: 10.3389/fnsys.2013.00008 (PMC3652307; doi:10.3389/fnsys.2013.00008)

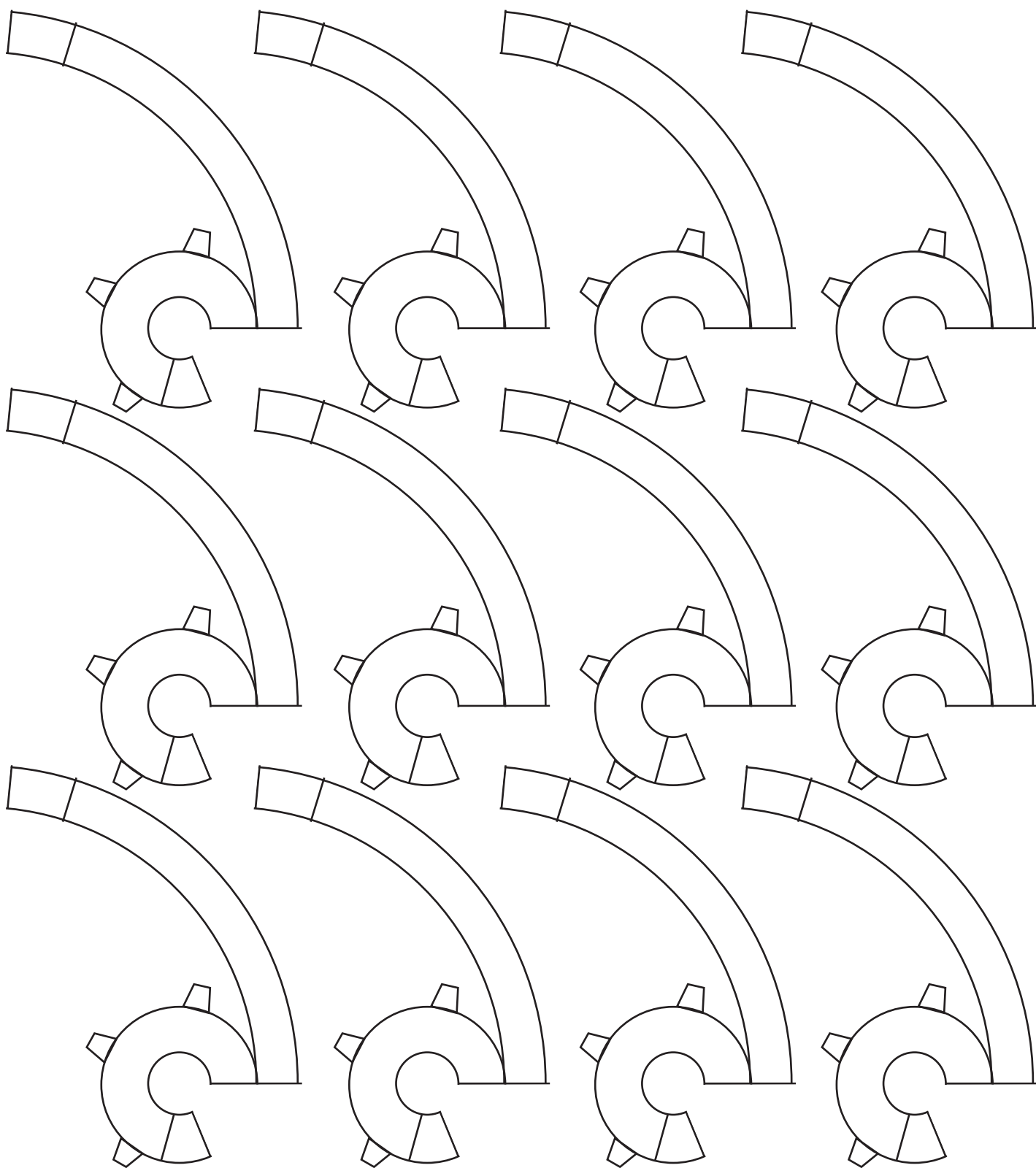

**flexDrive**

shield template

<https://github.com/open-ephys/flexDrive>

Supplement: Supplementary file 1 [file DataSheet1.ZIP › flexDrive_source_files_mar11/shield_cap/flexDrive_shield_cone.pdf]

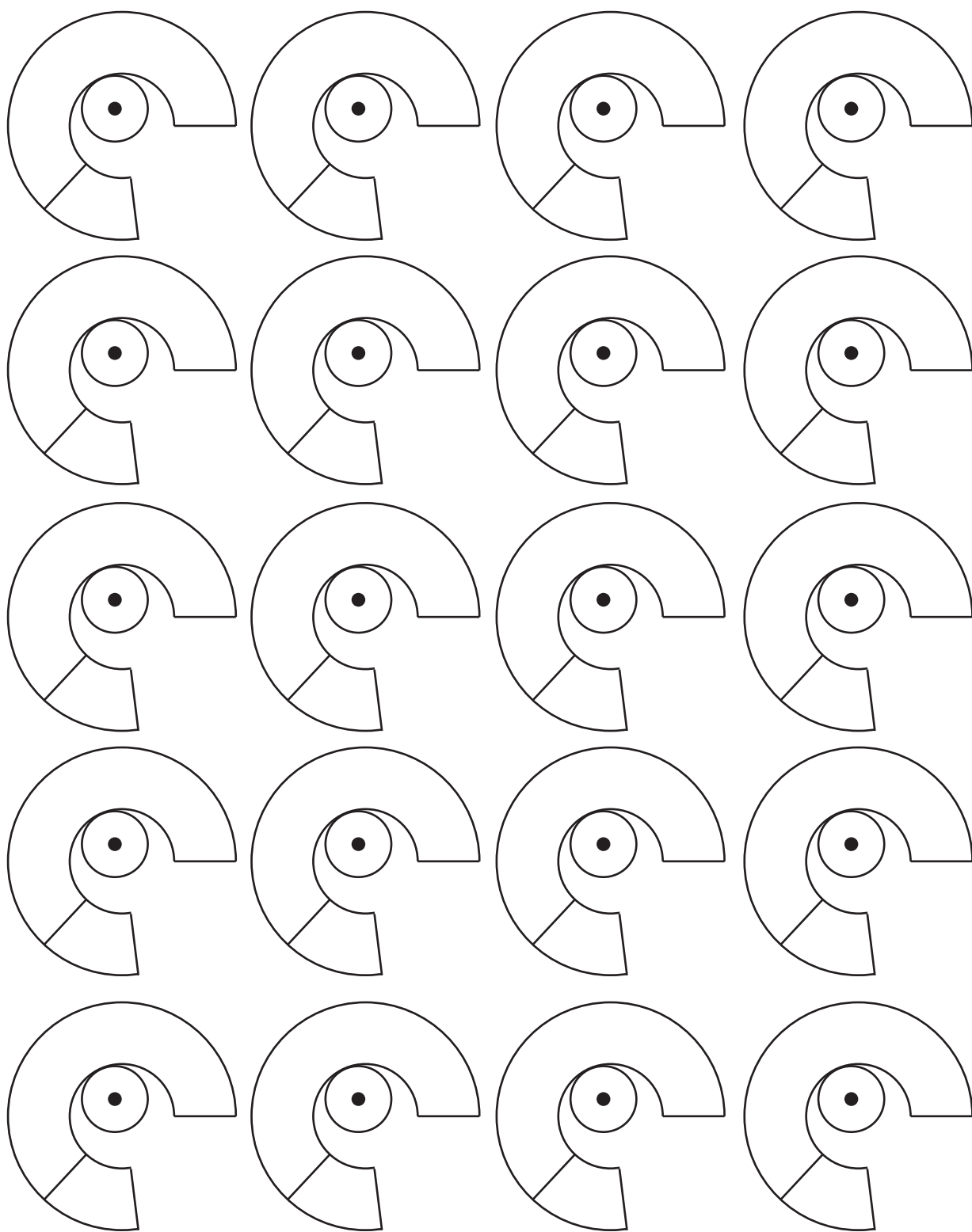

**flexDrive**

cap template

<https://github.com/open-ephys/flexDrive>

Supplement: Supplementary file 1 [file DataSheet1.ZIP › flexDrive_source_files_mar11/shield_cap/flexDrive_cap.pdf]
