# Supplementary material for: The flexDrive: an ultra-light implant for optical control and highly parallel chronic recording of neuronal ensembles in freely moving mice
Source: Front Syst Neurosci. 2013 May 13;7:8. doi: 10.3389/fnsys.2013.00008 (PMC3652307; doi:10.3389/fnsys.2013.00008)
Supplement: Supplementary file 2 [file Presentation1.PDF]

Materials and Methods
(supplementary)

Materials

All design files as well as detailed building instructions are available on the Moore lab website (http://neuroscience.brown.edu/moore/) and on Github (http://github.com/open-ephys/flexDrive). Where listed, sources are suggestions of companies that we previously purchased materials from.

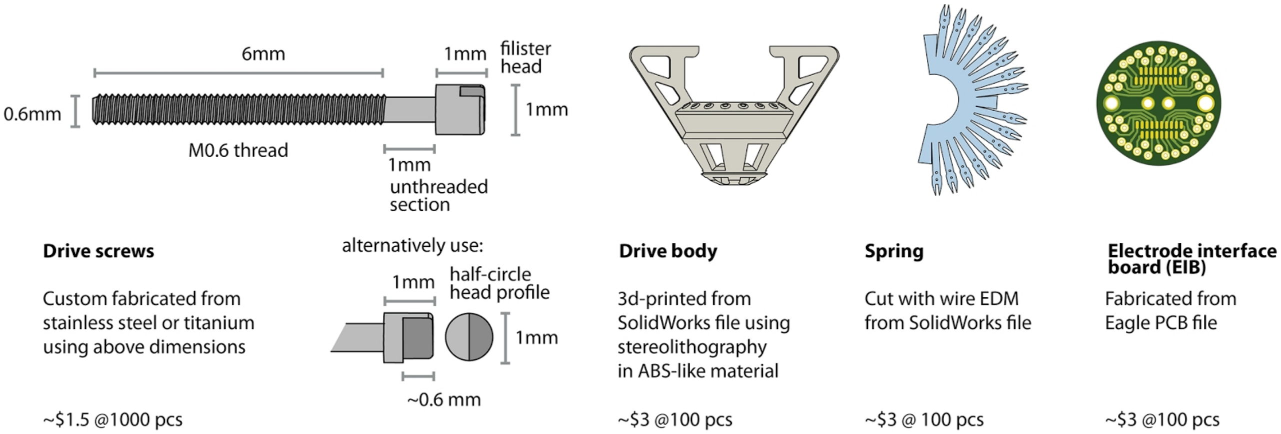

Supplementary Figure 1 Custom parts used in the design of the flexDrive.

Custom parts

16x Drive screws (custom M0.6x7mm from Antrin Miniature Specialties, Inc.) (Stainless steel or titanium, 6mm threaded length, 1mm under the head non-threaded, Filister head, 1mm height, 1mm diameter, 0.5mm slot depth. Alternatively: Half-circle head profile, 1mm height, 1mm diameter 0.6 mm cutout depth)
Drive body (3D printed, ABS like plastic, approto.com, design available online)
Steel spring (1095 spring steel, 0.002”, machined with wire EDM (Electric discharge machining), design available online)
Electrode interface board (EIB) (Neuralynx, or custom Printed circuit board (PCB), design available online)

Shielding / Enclosure

Overhead projector transparencies
Aluminum foil (for making the shielding & Cap, templates available online)
Cap from optic fiber ferrule (for attaching drive cap to drive)
Steel wire for grounding (Stainless Steel, Teflon (Dupont Teflon(r) PFA) coating, ~0.003" ,A-M Systems)
32x Gold pins (large, Neuralynx)

Connectors

2x Amplifier connectors (Omnetics or Molex)
or any other custom connector, depending on the choice of EIB

Polyimide tubing (Smallparts.com)
Shuttle tubes - 37 or 38 gauge
Guide tubes - 33 gauge
Stabilizer tubes- 26-28 gauge
Additional polyimide tubing for holding drive bottom assembly (for example 22 and 21ga) or stainless steel cannula depending on drive bottom layout

Optical fibers
Optical fiber, 125, 200 or 300µ core diameter (Thorlabs)
1.25mm OD ferrule connector (steel or ceramic, Precision Fiber Products)
Fiber polishing supplies
Alternatively: Ready to use fiber ferrule (for examples see Doric lenses or Thorlabs)

Electrodes
See protocols for tetrode or stereotrode fabrication (Nguyen et al. 2009)

Glue
Cyanoacrylate super glue (medium viscosity)
Epoxy (2 component, 5 minute epoxy)
Conductive epoxy for connecting ground wire to drive cone

Tools
Ceramic tipped forceps (Dumont A275B, FST)
Serrated scissors
Dumont #5 Forceps
Measuring calipers
Alligator clip stand for holding drive during assembly
Screwdriver for M0.6 screws (can be modified with guide cannula to prevent slipping)
Razorblades
Fine sandpaper (200-400 grit)
Small sharp scissors for cutting polyimide tubes
2 hemostats for holding the springs while soldering
Wire cutter for ground wires
Needle nose pliers for inserting gold pins
Soldering Iron (w/ solder, flux, etc)
Drill bit (#77) and pin vi

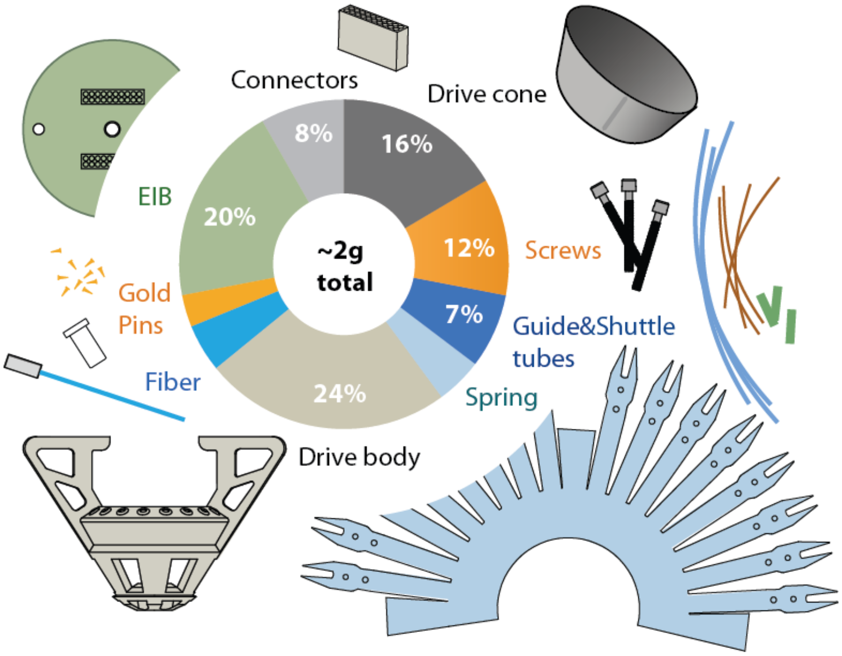

Supplementary Figure 2 Components of the flexDrive, and their relative contribution to the total implant weight.

Building instructions

Prepare electrodes

Use any suitable protocol (Nguyen et al. 2009; Gray et al. 1995; McNaughton et al. 1983)

Prepare EIB

- Either use commercial EIB provided by the maker of your electrophysiology system,
- Or if using custom PCB, solder connector to EIB and secure with epoxy. Test connections.

Prepare spring

- Clean and degrease the spring with ethanol or acetone.
- Bend spring into cone shape and align soldering tabs. The 'ears' on both end of the spring have to be perfectly aligned. This is accomplished relatively easily by clamping both ends of the spring with a hemostat and carefully shifting them until they are aligned.
- Hold in place using a hemostat or other clamp. Solder using an acid based flux, work fast to avoid overheating and breaking the spring.
- Remove all residual flux with ethanol.
- If too much solder is on the spring, it can be removed easily using a razor blade and/or fine sand paper. If possible, don't use de-soldering braid or any method that heats up the solder as this will likely change the alignment or break the spring.

Prepare shield and cap

- Print templates on standard overhead transparency sheet
- Glue aluminum foil to transparency sheet with epoxy for the cone part, but use plain transparency sheet for the cap part.
- Cut out parts using scissors, and glue with epoxy, clamp the parts with a hemostat while the epoxy cures.
- For cap: attach optical ferrule cap (different depending on EIB type) to cap. Alternatively, use small pieces of electrical tape to attach cap to drive after implant.

Prepare drive body

- Clean printed drive body: check for extra plastic on the inside fins and guides
- Depending on the quality of the 3d-print, finish the screw holes with #77 drill
- Clean off plastic dust with water, be careful when using ethanol as it will weaken the plastic when exposed for too long
- Carefully 'tap' screw holes by screwing a drive screw all the way in, and removing it from each hole. To speed up this step, solder a screw to a piece of metal to use as handle.

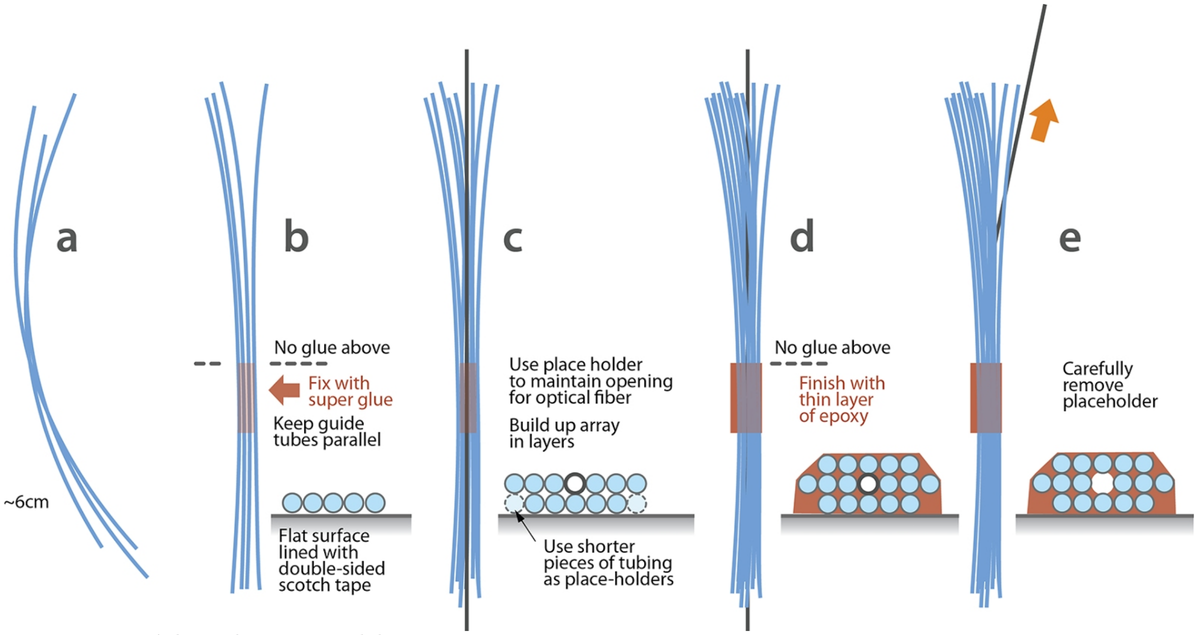

Prepare guide tube assembly

(Assembly steps that are illustrated are numbered a-l, optional steps are marked)

- Cut 16(or fewer) segments of 33ga polyimide tubes to around 6 cm length
- (a) Either glue the guide tubes into rows with superglue and arrange rows to form linear arrays (a-e)
- Or (not shown) bundle the tubes into a larger polyimide tube (we use 22ga) or steel cannula by adding more tubes one at a time and gently rearranging them.
- Make sure that no glue gets into the tubes, and that the tubes are free from glue above a consistent depth (b) so they are free to fan out at that depth.

- (c) To add a fiber to the array later, coat a piece of fiber or a steel cannula of the same diameter with mineral oil, and arrange into array like a guide tube. This will function as a place holder. Alternatively, add a larger diameter polyimide tube that can house the fiber later.

- (d) After the polyimide tubes are arranged into the desired pattern, fix them with a thin layer of epoxy. For guide tubes that are not supported by a layer of tubes from below (c), add shorter lengths of tubing to prop them up. After the epoxy has cured, (e) remove the placeholder for the fiber.

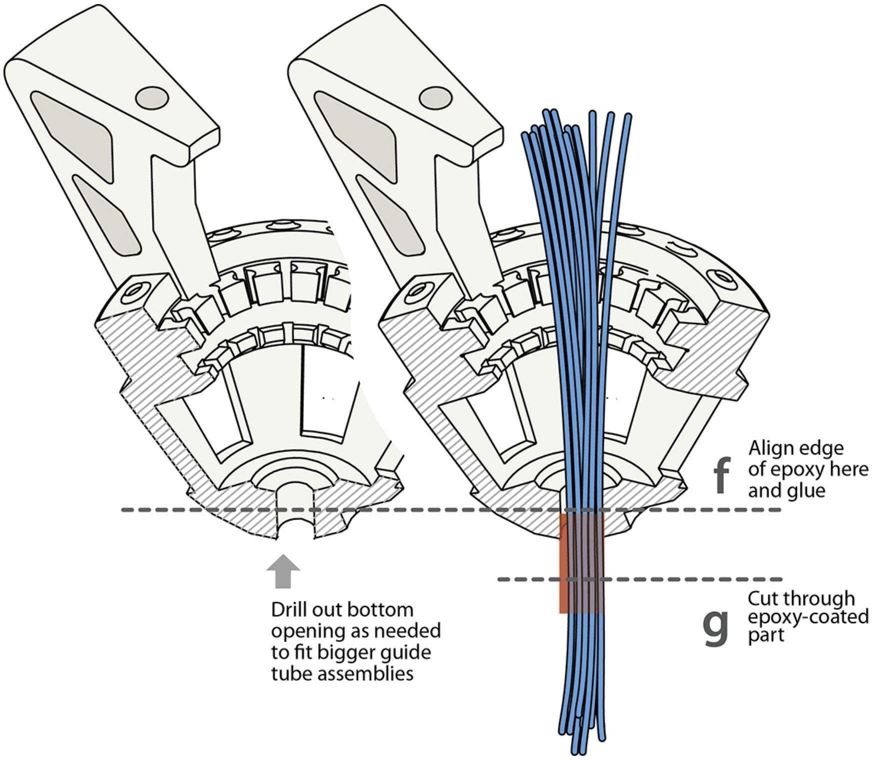

Assemble drive

- Glue the spring to the drive body using epoxy. Make sure the bottom and sides are making full contact to the drive body. Make sure that the spring is precisely aligned with the drive body (the small protrusions on the drive body should fit into the cutouts on the solder tabs of the spring).
- Let the epoxy cure fully before bending the screw arms. Otherwise, the tension of the spring can slowly stretch and detach the epoxy.
- (f) Glue guide tube assembly into drive bottom with epoxy, make sure the guide tubes are aligned. For most applications the tubes should be parallel to the main axis of the drive, but deviations of up to 15deg. are possible if desired.
- (g) Cut the guide tube assembly about 5mm from the drive bottom using a fresh razor blade. The length of the protruding guide tubes can be chosen to fir the experimental design: Longer lengths increase visibility during the implant surgery and are useful in order to leave space for head posts etc., but shorter lengths lower the center of gravity on the head.
- (h) Move guide tubes into the row of slots at the insider perimeter of the drive body. Make sure to keep the tubes as straight as possible.
- (i) Hold guide tubes in place by sliding short pieces of 26ga polyimide tube ('stabilizer tube)) over the guide tube.
- (j) Glue guide tubes and their stabilizer tubes into place with thin layer of epoxy.
- (optional) Map out the relationship between guide tubes at the drive bottom and the drives by inserting a thin wire into the guide tubes one at a time.
- Cut the guide tubes so that they extend about a mm above the rim of the driv body.
- Screw in the screws about half way, and bend and slide the spring arms under the screw heads using forceps.
- Cut 16 pieces of 37 or 38 ga polyimide tubing to ~2cm to make the shuttle tubes.
- (k) Insert shuttle tubes into guide tubes so that they are inserted ~1mm at the most retracted

position.

- (l) Glue shuttle tube to spring arm, use two layers of epoxy for added stability.
- Cut the shuttle tubes tubes a few mm upwards from where they meet the springs.
- Cleave optical fiber to desired length, attach to ferrule and polish both ends.
- (or - if using fibers with prepared ferrules), cut to length and polish bottom end.
- (optional) cut a groove into the ferrule using a high-speed cutting wheel to improve adhesion of epoxy.
- Insert optical fibers into openings in the guide tube array, don't glue them yet.
- Attach EIB to the drive body with screws and/or epoxy, at the same time, insert fiber/s through the holes in EIB.
- Position fibers at desired depth, and fix them to the EIB with epoxy. Make sure to add epoxy to the top and bottom of the EIB to securely stabilize the ferrule.

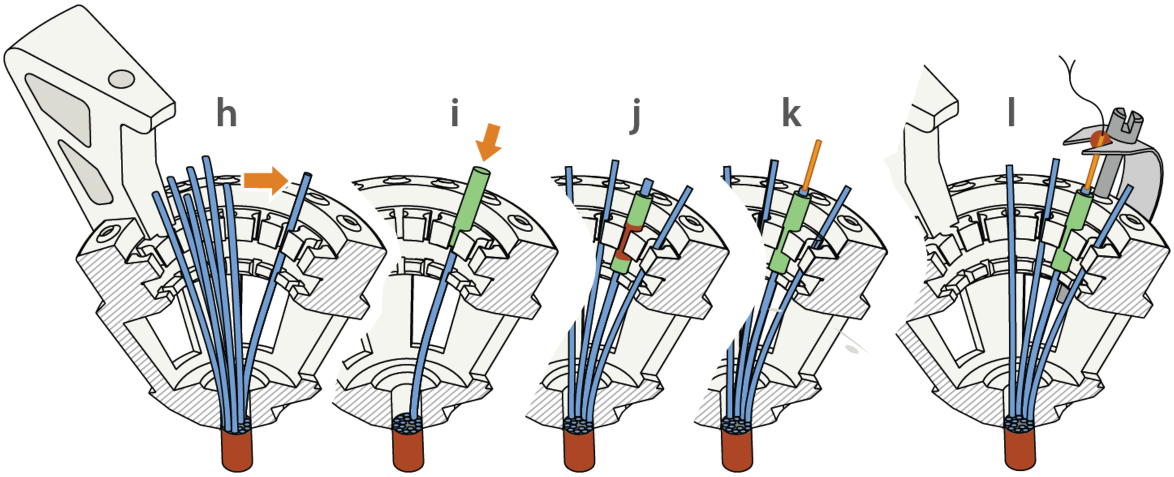

- (l) Load electrodes into the shuttle tubes using ceramic tipped forceps.
- Follow protocol for electrode loading appropriate for your EIB (see Kloosterman et al. 2009 for an example)
- Cut each electrode to the desired length with very sharp scissors, if required, make small adjustments to electrode depth, and glue it to the top of shuttle tubes with epoxy. After epoxy is cured, connect electrode to EIB using gold pins and/or solder.

- Attach ground and shield wires to the EIB with gold pins and/or solder. The ground wire should extend to the bottom of the drive with enough free length to reach the desired ground site during the implant surgery.

- Push the ground wire through the bottom opening of the protective cone and attach cone with epoxy. Make sure there is a strong bridge of epoxy between the side of the drive body and the cone. Use multiple layers of epoxy if needed. Take extra care not to touch and bend electrodes with the protruding end of the ground wire.

- Use conductive epoxy to connect the shield wire to the drive shield. Secure with additional layer of epoxy.

- Apply small amount of mineral or silicone oil to the springs where they meet the screws.

Now the drive is ready to store until the implant.

- Prior to implanting, electroplate the electrodes (optional), and measure the electrode impedances.
- Fill the guide tubes with silicone or mineral oil. Take care not to coat any other part of the drive with oil in order to ensure proper cement attachment during the surgery. Take notes of the exact electrode depth at the screw position that will be used at the time of implant.

Implant surgery

Perform the implant surgery according to the animal care guidelines of your host institution. This outline serves only as broad suggestions on how to handle some aspects of the surgery that are affected by the drive itself and are not directly applicable to any specific use of the drive implant.

- Depending on the method used to connect the ground wire to the grounding site, verify that all connectors function properly. Some types of connectors such as mill-max pins have a lot of friction on first use

- Make sure that all electrodes are at well defined depths at the time of implant, so that the electrode position can be precisely reconstructed later

- Perform a craniotomy suitable for the size of the electrode array and the targeted recording site using a suitable method.

- We find that performing a durotomy minimizes brain deformation and yields better recordings at small electrode pitches.

- During the surgery, the drive can be attached to the stereotactic frame by clamping or gluing a headstage connector to the stereotactic frame and connecting it to the connectors on the EIB. If the drive is implanted with some electrodes already extended, make sure that these electrodes are perfectly aligned with the axis of the stereotactic frame that is used to lower the drive into the brain.

- During surgery, fill the space between the guide tube array and the brain with compatible surgical lubricant or mineral oil/paraffin according to the surgical protocol.

- Attach the drive to the skull using dental cement, skull screws and/or adhesives according to your protocol. Make sure that no adhesive gets under the guide tube array.

- If required, add an additional head-post for use in head-fixed recordings according to your protocol. Note that the drive body itself is not designed to withstand significant forces and should not be used to hold animals.

Electrodes can be lowered as soon as the animal has recovered from surgery. Delaying the onset of the lowering for more than ~5 days post surgery increases the risk that dura and bone regrowth interferes with the electrodes.

We recommend housing all implanted animals without cage mates in order to avoid chewing of the

implant and the wound margins. Further, food grates or similar features of the cage that could catch parts of the drive implant should be removed. This is especially important if additional protruding implants such as head-posts are used.

Post-mortem localization of recording sites can be accomplished either by histologically localizing electrolytic lesions (Bragin et al. 2000; Fee et al. 2001; Jog et al. 2002) or by localizing the electrode track by staining for markers of microglia or macrophages (Freire et al. 2011).
